# Rethinking Cochlear Implants: The Need for Precise Temporal Coding

**DOI:** 10.1002/advs.78601

**Published:** 2026-11-10

**Authors:** Nicole Rosskothen‐Kuhl, Jan W. H. Schnupp

**Affiliations:** ^1^ Neurobiological Research Laboratory, Section for Experimental and Clinical Otology Department of Otorhinolaryngology Faculty of Medicine Medical Center – University of Freiburg Freiburg Germany; ^2^ Bernstein Center Freiburg and Faculty of Biology University of Freiburg Freiburg Germany; ^3^ Gerald Choa Neuroscience Institute Chinese University of Hong Kong Sha Tin Hong Kong SAR China; ^4^ Department of Otorhinolaryngology, Head and Neck Surgery Chinese University of Hong Kong Sha Tin Hong Kong SAR China; ^5^ School of Biomedical Sciences Chinese University of Hong Kong Sha Tin Hong Kong SAR China

**Keywords:** binaural hearing, cochlear implant, deafness, hearing experience, interaural time difference, pitch, prosthetics, temporal coding

## Abstract

Hearing relies heavily on the encoding of rapid temporal patterns, yet contemporary cochlear implants (CIs) largely neglect precise temporal coding in favor of strategies that prioritize envelope information and minimize channel interactions. Here, we review the physiology of temporal coding in normal hearing, the limitations of current CI strategies, and emerging evidence that better temporal coding could substantially improve outcomes. Contemporary CIs face three major limitations: poor intensity coding, coarse place coding with only a modest number of resolvable frequency channels, and critically, the absence of precise pulse timing codes to support pitch perception and binaural hearing. Human bilateral CI patients generally show much worse interaural time difference (ITD) sensitivity than normally hearing individuals. However, rats fitted with bilateral CIs are no less sensitive to ITD than normally hearing rats or humans. These animals rely heavily and specifically on pulse timing cues for binaural hearing, with time‐intensity trading ratios around 20 µs/dB. We argue that prolonged exposure to random or misleading pulse timing from clinical processors may cause maladaptive suppression of innate ITD sensitivity. Developing novel coding strategies that exploit the auditory system's remarkable sensitivity to precise temporal patterns represents a promising but challenging path toward truly rich prosthetic hearing.

## Graphical abstract

Spatial hearing relies on sensitivity to interaural temporal cues, but this sensitivity is often poorly developed in patients with cochlear implants, particularly those with early‐onset hearing loss. Rats with cochlear implants, however, demonstrate excellent temporal sensitivity, suggesting that this limitation is not inevitable. Preserving temporal information in the pulse timing of cochlear implant stimulation could improve spatial hearing.

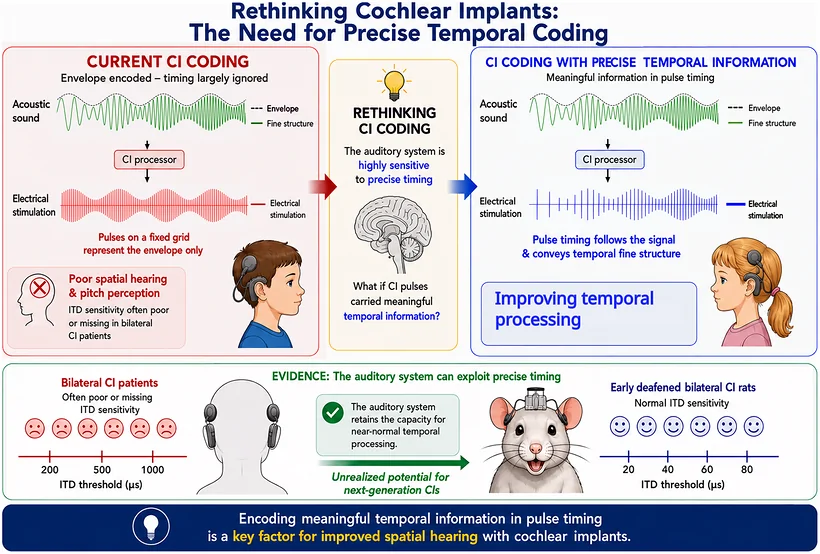

## 1 Introduction

Fundamentally, the sense of hearing is all about time and timing. More so than other senses such as vision or touch, the auditory system is exceptionally well adapted to detect and interpret very fast temporal patterns. Sound consists of tiny, and usually extremely rapid, fluctuations in air pressure. Although these pressure changes are physically very small, their precise temporal patterns provide a treasure trove of information about the environment around us. The auditory system must interpret these temporal patterns with great temporal precision in order to be able to infer properties of sound sources, such as their identity, location, and movement.

The first step in the physiological processes of hearing is the encoding of the temporal patterns of sound into patterns of neural activity, a function performed by the cochlea in the inner ear. Sound is transmitted through the external auditory canal, eardrum, and middle ear ossicles to the cochlea, where it is separated into different frequency components by a mechanical filter bank which is formed by mechanical interactions between the basilar membrane and the surrounding fluid columns. The basal portion of the basilar membrane, closest to the middle ear ossicles, is mechanically stiff and coupled to the shortest fluid column with a relatively small effective mass, causing it to resonate at high frequencies. More distant parts of the basilar membrane become increasingly less stiff and are associated with longer, and therefore heavier, fluid columns, and they therefore resonate at increasingly lower frequencies. Vibrations of the basilar membrane are then encoded as neural signals by extremely sensitive sensory hair cells positioned along the basilar membrane, and these hair cells synapse in a spatially precise manner with axons of the auditory nerve (AN). AN fibers are therefore “tuned” to systematically different sound frequencies, resulting in a neural *place code* for the spectral content of sound. This place coding is often referred to as “tonotopy”, and while this no doubt plays an important role in the neural representations of sound, it is only part of the story. AN fibers are also able to synchronize the timing of their action potential firing to the temporal fine structure of incoming sounds with remarkable accuracy. This creates a *spike timing code* which operates alongside the tonotopic place code to convey a great amount of auditory information from the ear to the brain. We will look at this temporal code in more detail below.

The whole system is a marvel of biophysics, which depends on the precise interplay of a great number of delicate structures and processes in the ear. Among the most remarkable of those structures are undoubtedly the cochlear sensory hair cells. To be able to detect even very quiet sounds, they must be sensitive enough to respond to mechanical vibrations as small as a nanometer, but also fast enough to send signals through their synapses to AN fibers with microsecond precision. Unfortunately, it appears to be inevitable that structures which are so phenomenally sensitive and precise must also be fragile. Indeed, there appear to be countless ways in which the sensory hair cells of the cochlea can be damaged, from excessive noise exposure to toxic side effects of certain medicines and chemicals, to genetic defects. Hair cells may also simply “wear out” as we age, leaving us with age‐related hearing loss. In mammals, hair cells that are lost do not regenerate spontaneously. Although recent studies have reported encouraging progress toward hair cell regeneration in animal models [1, 2], the technical hurdles involved in turning this research into routine clinical practice remain formidable, and it would be unrealistic to expect regenerative therapy for cochlear hair cell loss to become widely available in the coming years. If we lose only a small portion of our hair cells, we may be able to compensate for the resulting modest loss in auditory sensitivity, but extensive loss of hair cells is by some margin the most common cause of severe or profound hearing loss. In human society, where spoken communication is often a key element of most social interactions, such severe‐to‐profound hearing loss is highly debilitating.

Deaf communities have reacted to the challenges of severe hearing loss by developing highly sophisticated and effective sign languages, which can express the full spectrum of human ideas and emotions. They often had to do so with little support, and sometimes outright opposition, from their normally hearing peers. For example, a highly influential International Congress for the Education of the Deaf held in 1880 in Milan, at which almost all participants were normally hearing, voted to strongly discourage the teaching and use of sign languages in deaf education [3]. Instead, they expected deaf children to succeed in life by directly tackling the incomparably harder task of becoming expert lip readers. Unrealistic expectations and misguided initiatives based on a poor understanding of the needs of deaf children were pervasive through history until relatively recently. According to the World Federation of the Deaf, more than half of the world's nations still do not give official, legal recognition to sign languages, and most of the ones who do only introduced such laws in the 2000s.

Given this historical record, it is understandable when some deaf communities react with skepticism whenever normally hearing authorities claim to know what is best for them. They are also often rightfully proud of the remarkable cultural achievement that the development of sign languages represents. However, only a very small proportion of humans are competent users of sign languages. Accurate estimates are rare, and range from 0.23% for British Sign Language use in Scotland [4], to as high as 2.8% for American Sign Language use in the USA [5]. The reach of sign languages is further reduced by the fact that there are, according to online reports, as many as 300 or more mutually incomprehensible sign languages globally, although precise numbers are hard to find. Thus, despite their linguistic sophistication, sign languages are just not widespread or unified enough to offer deaf patients the easy access to hundreds of millions of potential conversation partners that people who can understand one of the world's major spoken languages take for granted. Fortunately, for many deaf patients, restoring some auditory capabilities through neuroprosthetic cochlear implants (CIs; Figure 1) has become a valuable option. Indeed, at the time of writing, well over one million patients worldwide have been fitted with CI devices [6], and many of them derive enough benefits from their implants that they can learn to have conversations on the telephone, attend mainstream schools, and otherwise operate successfully in a world of spoken communication.

**FIGURE 1:**
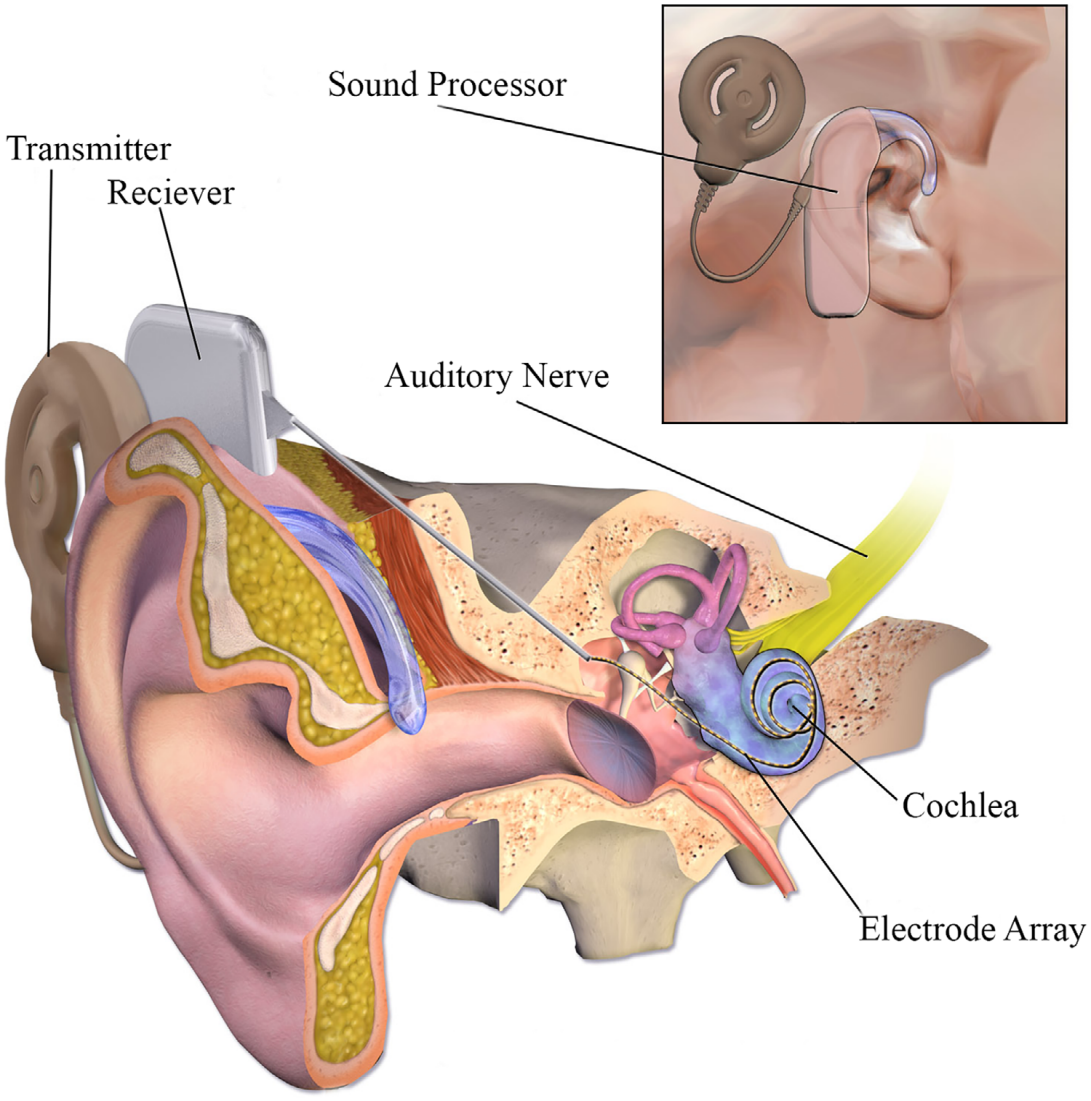
The cochlear implant (CI). Schematic representation of a cross‐section of the peripheral auditory system of a CI patient, showing the external and internal components of a CI device. Top right: CI components visible from the outside, consisting of a sound processor and a transmitter. Figure adapted from [7].

But although CIs already offer very significant benefits to many patients, the quality of the hearing experience they provide still falls far short of that offered by a healthy, “normal” cochlea. CIs comprise a small electrode array which is inserted into the inner ear, typically into the scala tympani, such that electrode contacts come to lie adjacent to the tonotopic array of AN axons (Figure 1). Electrical pulses sent down the electrode array bypass the mechanical filtering and transduction processes that would normally be provided by the basilar membrane and the cochlear hair cells to activate AN fibers directly. The fundamental design is, in a sense, very simple: by placing stimulation electrodes at different points along the length of the basilar membrane, CIs tap into the place code for frequency. That this works well enough to permit speech recognition in most users is quite remarkable, and the simplicity of the approach is in some respects also one of its strengths: to be genuinely useful, and to warrant the significant discomfort, risk and expense that comes with the surgical implantation and audiological fitting of these devices, CIs have to be able to operate reliably and consistently for many years. Simple and robust designs have fewer points of failure. Still, there are many aspects of the physiology of “normal”, acoustic hearing that contemporary, relatively simple CI designs cannot replicate. The limitations of contemporary CIs in encoding of sounds fall into three distinct categories: reduced intensity coding, poor place coding for sound frequency content, and poor temporal fine structure coding for pitch and binaural hearing. Let us consider each of them briefly in turn.

## 2 Intensity Coding Limitations of Contemporary CIs

If we take the view that hearing operates primarily through a tonotopic place code, then the number of sounds that this place code can distinguish will be limited both by the number of frequency channels that the place code can resolve, as well as by the number of intensity steps that can be discriminated within any one frequency channel. Unfortunately, as we are about to see, CI hearing is quite limited in both frequency and intensity resolving power, and it is not obvious that either of them can be easily improved. For starters, prosthetic hearing via CIs suffers from a very limited dynamic range when compared to normal acoustic hearing. The normal auditory system is capable of operating over an extremely wide range of sound intensities. The loudest sounds that people can tolerate carries 120 dB, which is 1000 000 000 000 times more sound energy than the faintest sounds that people can just about detect. Most of the mechanisms that make this extremely wide dynamic range possible are associated with cochlear hair cells. These include non‐linear, sound‐level‐dependent amplification of weak signals by electromotility in outer hair cells, as well as sets of inner hair cell‐AN fiber synapses of varying sensitivity. These synapses give rise to sets of nerve fibers that differ in the range of sound intensities they can optimally encode. All these biological mechanisms are absent in CI hearing, which relies on direct electrical stimulation of nerve fibers, and which is fundamentally somewhat binary in nature. A stimulus current pulse is either strong enough to trigger action potentials in nearby nerve fibers, or it is not. The extent to which it is possible to “dose” current pulses such that pulses of increasing amplitude drive a gradually larger number of nearby nerve fibers is quite limited. Consequently, it is unsurprising that the dynamic range of current intensities that CI users can operate over has been found to be only a very modest 8–20 dB [8, 9, 10], which means that the intensity dynamic range for CI stimulation is roughly *10–100 billion times* narrower than that of normal hearing. Modern CIs do their best to compensate for this limitation by incorporating automatic gain control (AGC) circuits, which dynamically change how the amplitude distributions of incoming sounds are mapped onto electric stimulus pulse amplitudes. By and large, AGC seems to work quite well, but it cannot overcome the fundamental issue that any efforts to map a range of acoustic intensities onto electric ones will incur a loss of information if the number of discriminable intensity steps in electric hearing is smaller than that in normal hearing. Chua and colleagues [11] and Nelson and colleagues [12] observed that the number of discriminable intensity steps in CI users varied greatly between patients, ranging from ∼ 5 to ∼ 45, but it was always substantially smaller than the 83 discriminable steps seen in normal acoustic hearing.

## 3 Place Coding Limitations of Contemporary CIs

A major limitation of CI hearing that is much harder to work around is the poor spatial resolution with which place coding for frequency can operate in contemporary CIs. The normal human ear has approximately 3500 inner hair cells, meaning that it, in a sense, samples the tonotopic place code for sound frequencies at 3500 points along the basilar membrane. However, it would be inaccurate to think of these as 3500 “frequency pixels”. While 3500 photoreceptors on a retina would be able to independently sample 3500 distinct points of visual space, neighboring inner hair cells provide highly overlapping rather than independent samples of a sound's frequency spectrum. The mechanical filtering of sounds by the basilar membrane is not that sharp, and neighboring points on the basilar membrane are physically coupled, which reduces their ability to resonate independently to different frequencies. Indeed, the number of *independent* frequency bands that the human inner ear can distinguish based on place coding alone, the number of so‐called “critical bands”, has been estimated to be only in the order of 40 or so [13]. Curious readers who are not deeply familiar with the intricacies of auditory physiology may be surprised by how low this number is. A typical grand piano has 88 keys on its keyboard, more than twice as many as the human ear has critical bands. Nevertheless, we find it very easy to distinguish the change in the fundamental frequency, or pitch, of musical notes that differ by one semitone step (one key up or down on the piano). In fact, the smallest pitch step that people can typically discriminate is smaller than one‐tenth of a semitone (< 0.6% change in frequency) [14], and the number of pitches that the normal human ear can discriminate may be as high as 1400. What these numbers illustrate is that even very simple, fundamental questions such as “how good is the normal human ear at distinguishing frequencies”, or, “how does the human auditory system achieve frequency discrimination”, do not have simple answers. Much depends on the specifics of the auditory task or phenomenon one is trying to explain, and there is much we still don't know about how the auditory system creates the sensation of different pitches and spectral timbres. Also, many researchers believe that tonotopic place coding alone can not provide all the answers, and that the spike timing codes mentioned briefly earlier also play an important role. For a more detailed treatment of these issues, see [15].

In any event, regardless of whether we take the number of distinguishable “frequency channels” in the normal human ear to be as low as around ∼ 40 critical bands or as high as ∼ 1400 pure tone frequency “just noticeable differences” (JNDs), what is certain is that the number of frequency bands available to CI users is much lower. Contemporary CIs have only between 12 and 24 independent stimulating electrode sites. Given the sophistication of modern electronics, one might think that it ought to be fairly easy to substantially increase that number of electrodes, and thereby offer a larger number of “frequency input channels” to CI users. However, the main bottleneck here is not technology, but rather it is the physiology of the electrode‐nerve interface. Contemporary CI electrode arrays are inserted into the scala tympani of the cochlear duct, where they lie close, but not directly adjacent, to the AN fibers they are aiming to stimulate, and the electrode contacts sit in the perilymphatic fluid that fills the scala tympani. Like most physiological fluids, perilymph is rich in dissolved electrolytes and therefore a good conductor of electric currents. Stimulus currents sent out through any one CI electrode can therefore travel not just to the closest AN fiber, but also easily spread sideways toward neighboring electrodes and more remote AN fibers. The electrical stimulus is not well confined to a narrow region, which creates “channel cross talk” and “channel interactions” that substantially limit the precision with which CIs can target the tonotopic place code. Indeed, psychoacoustic experiments have shown that the number of discrete tonotopic channels that CI users can reliably discriminate is often as low as eight [16, 17]. More recent studies [18, 19], however, have demonstrated that improved electrode positioning with perimodiolar arrays may increase the number of effective spectral channels to perhaps as many as 12 to 16. This is inferred from modest improvements in speech recognition when the number of active channels made available to patients is gradually increased.

Nevertheless, current spread and channel interactions continue to severely limit the spatial resolution with which contemporary CIs can tap into the cochlea's place code for sound frequency, and this thought to be one of the main reasons why the quality of prosthetic hearing remains substantially worse than that afforded by normal, healthy ears. It is likely to be a leading contributor to the poor perception of musical melody and pitch afforded by Cis [20, 21, 22, 23, 24], as well as the difficulties that most CI patients face when listening to tonal languages where a change in pitch of a word can totally change its meaning [25, 26, 27]. In fact, it is perhaps surprising that as few as eight to twelve independent frequency channels are usually enough to enable CI users to learn to understand speech through their devices at all. However, may explain why most CI patients need learning periods of many months to be able to make good use of this spectrally extremely impoverished input [28, 29, 30].

Unsurprisingly, many efforts have been taken to try to improve on the poor place coding in CIs. These range from “current steering”, which attempts to focus current patterns through patterned activation of multiple electrode channels [31, 32], to radical redesigns of the implant‐nerve interface which would rely on optogenetics to make AN fibers light sensitive and then use LED array implants instead of electrodes to provide a tonotopically more focused activation of the AN [33, 34]. However, the technical obstacles to these approaches are formidable, and in the opinion of these authors, the next major breakthroughs in CI technology are more likely to come from more sophisticated temporal patterning, rather than from improved spatial resolution, in the stimulation patterns delivered by CI devices.

## 4 Temporal Coding Limitations of Contemporary CIs

To understand why the next “low hanging fruit” in CI development may well be improved temporal coding, we should first remind ourselves of quite how much detailed and precise information AN fibers encode through temporal, rather than spatial, discharge patterns. This facet of natural hearing has been underappreciated, and, in our opinion, has been inadequately considered in the design of sound encoding strategies that have prevailed in the clinical device market for over three decades. Few datasets illustrate this more beautifully than the one by Cariani [35] reproduced here in Figure 2. That figure shows, in histogram form, how the firing of a set of 42 AN fibers recorded from a cat is modulated by the presentation of an “artificial vowel sound”. The artificial vowel used here is a simplified version of the types of sounds that we use in speech all the time, and it consists of a periodic pulse at a rate, or “fundamental frequency”, of 80 Hz, which would give it a low pitch roughly equivalent to the note E2 on a piano or the voice of an adult male speaker speaking with a low, relaxed tone. The 80 Hz pulses excite a 640 Hz resonance (or “formant”), just like the resonance that our vocal tract would make when we shape our mouth to say “oh”. Based on this information, if you had to guess how the AN would encode this “oh” sound, with its 80 Hz fundamental and its 640 Hz formant, and your knowledge of the auditory system was limited to a naive understanding of tonotopic place coding for frequencies, then you might naturally assume that this “oh” sound would excite AN fibers tuned to 80 Hz and fibers tuned to 640 Hz, and the rest of the AN fibers would hardly fire at all. Figure 2 shows that this reasonable guess would be way off the mark. In Figure 2B, AN fiber responses are arranged according to the frequency they are tuned to, from neurons tuned to about 200 Hz at the bottom to frequencies above 10 000 Hz at the top. *All of them* respond to the artificial vowel. Even 10 000 Hz fibers, which are tuned to frequencies a full four octaves away from the formant, and six octaves away from the fundamental frequency, are clearly excited by this low pitched “oh”. Even more importantly, *all* AN fibers, irrespective of their position in the tonotopic frequency place code, faithfully reflect features of the temporal pattern of the sound stimulus in their own temporal firing patterns. Thus, for example, the observation that the most common inter‐spike‐interval in the discharge of the 10 000 Hz fiber is 12.5 ms gives us a strong indication that the pitch of this sound must be 1/0.0125 s^−1^ = 80 Hz. In fact, in this example, the temporal pattern code of the AN firing is arguably more obvious and easier to read than the place code.

**FIGURE 2:**
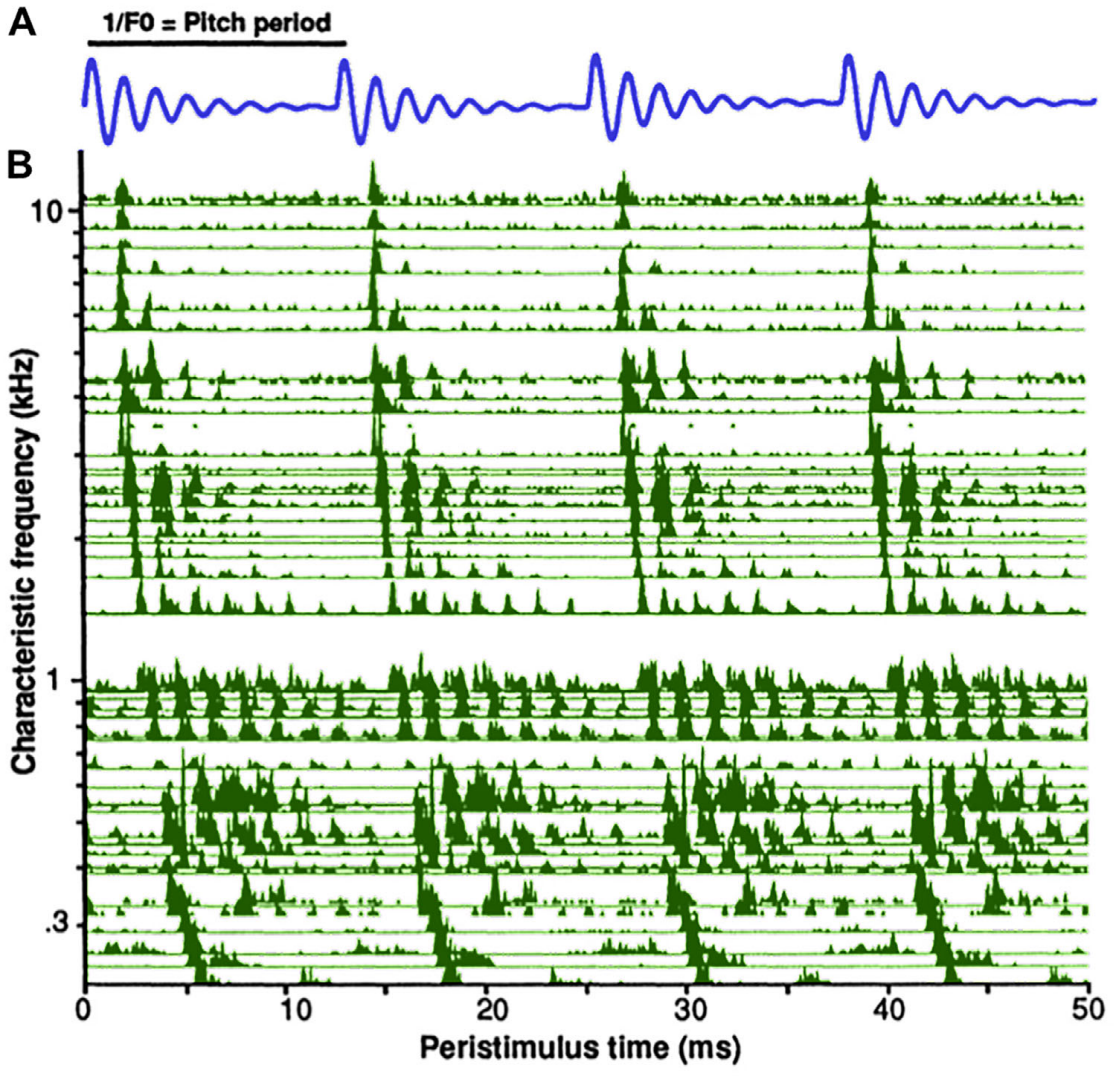
Recordings from cat AN fibers illustrate the wealth of information about incoming sounds which, in normal hearing, is encoded in the temporal patterns of AN fiber discharges. (A) An "artificial vowel” sound with a 12.5 ms period, corresponding to a fundamental frequency of 80 Hz and a resonance at 640 Hz is played into the ear of an anesthetized cat. (B) Post stimulus time histograms of 42 individual AN fibers, arranged by the characteristic frequency each fiber is tuned to, as shown by the axis to the left. Note that the regular, periodic temporal waveform of this sound is clearly reflected by the discharge patterns of the AN fibers, and that fibers across the entire tonotopic array, including those tuned to characteristic frequencies several octaves removed from the sound's fundamental or resonant frequencies, respond to this stimulus with discharges that “phase lock” to the temporal features of the sound. Adapted from Figure 4 of.

The auditory brain certainly seems to use the information that is encoded in these action potential timing codes, and it combines it with tonotopic place and firing rate coded information to determine, for example, what the pitch of a complex sound is, or, to tell where it came from. For the latter of these two objectives, the auditory brainstem compares the timing of AN discharges from the left and right ear to compute so‐called interaural time differences (ITDs), which are a powerful cue to sound source direction. If a sound arrives slightly earlier in the left ear, this indicates that the sound source is closer to the left ear than the right, and is therefore on the left side relative to the midline. ITDs are easy to manipulate in sounds presented binaurally over headphones, and psychoacoustic measurements of ITD thresholds have shown that they can be as small as 7–10 µs under optimal conditions [36, 37, 38, 39]. However, thresholds vary across normally hearing listeners depending on age, stimulus, and experimental paradigms. Based on other studies [40, 41, 42, 43], one might consider a “normal” (rather than optimal) human ITD threshold for acoustic hearing to fall in the range of 25–70 µs. ITD cues play a key role in sound source localization and contribute importantly to the spatial segregation of complex auditory scenes [44, 45]. The auditory brainstem can therefore analyze the timing of AN fiber discharges with astonishing precision. Whether the contribution of spike timing codes to pitch perception or other aspects of hearing are equally impressive is still a matter of debate, but there is some indication that the brainstem mechanisms for the processing of temporal pitch and ITD cues may be linked [46].

Fast and precise temporal patterning is certainly used by normal hearing, but working very fast and with very high temporal precision is easy for modern electronic circuits, so one might think that precise temporal coding would have been worked into CI coding strategies right from the start. Unfortunately, this is mostly not the case. For over thirty years, clinical CI processors have relied predominantly or entirely on strategies closely related to the continuous interleaved sampling (CIS) strategy first introduced by Wilson and colleagues [47]. A key design decision made by Wilson and his team was to try to prevent the already poor place coding in CIs to become further degraded by so called “channel interactions” [48], which can arise when temporally overlapping current pulses from neighboring electrode channels interfere. The simple solution they chose to prevent potential unwanted channel interaction effects was to decide that only one electrode channel would be active at any one time. Every electrode emits biphasic electric current pulses at a constant rate between 900 and 3700 pulses per second (pps) [49]. The pulses are brief, so each electrode is off most of the time, even when stimulation is active. There is a small delay in between the pulse in one electrode and the pulse in the next electrode (pulses are “interleaved”), and the amplitude of the stimulus pulses is then modulated to reflect (“sample”) the amplitude “envelope” of incoming sounds. Figure 3 illustrates how one such frequency band of a small snippet of a speech sound, centered at 925 Hz (Figure 3A), is encoded in this manner as an electric stimulus pulse train (Figure 3B). When we line up the original sound with the current pulse train that is meant to encode it (Figure 3C), then it becomes obvious that, although the changing pulse amplitudes capture relatively slowly evolving changes in sound amplitude envelope well, the timing of the pulses does not reliably or precisely line up with temporal features, such as peaks or troughs, of either the fine structure or the envelope of the incoming sounds. That should be unsurprising: the timing of the electric pulses is entirely independent of the incoming sound. But since AN action potentials will be triggered by the steep flanks of the electric pulse trains, the action potentials cannot align with temporal features of the sound stimulus with an accuracy that is better than ± half the pulse interval. At pulse rates of 900–1500 pps, the most commonly used pulse rate in clinical practice, this limits the accuracy of temporal coding in CIs to ±333 to ±555 µs, which seems inadequate if the aim is to support, for example, ITD discrimination thresholds that ought to be in the range of approximately 25–70 µs.

**FIGURE 3:**
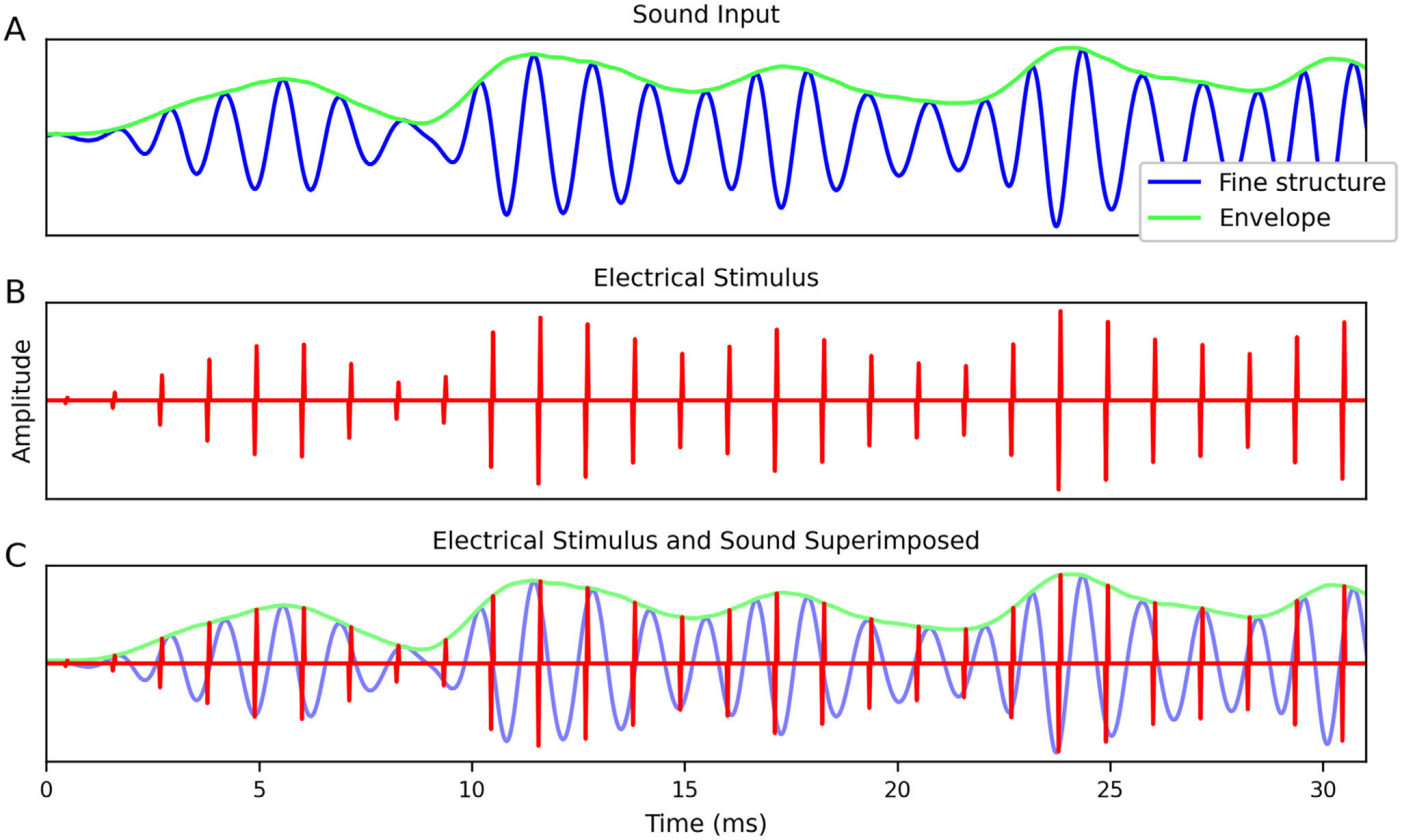
Processing principle of the prototypical coding strategy used in clinical CIs: Continuous Interleaved Sampling (CIS). (A) Incoming sounds are bandpass filtered, and for each band of the sound signal, the amplitude envelope (green) is computed. Note that the fine structure (blue) of the signal is discarded from further encoding of the stimulus sent to the corresponding electrode. (B) A constant train of biphasic electric stimulus pulses (red) with a fixed pulse rate (typically ∼ 900 pps) is amplitude modulated to reflect the envelope of the incoming sound (shown in A). (C) Comparing the electrical stimulus sent to the AN (red) against either the fine structure (blue) or the envelope (green) shows that the timing of the pulses does not reliably or precisely line up with any temporal features (maxima, minima, zero crossings) of either the fine structure or the envelope of the sound that the pulses are meant to encode. Only relatively slow changes in the amplitude of the sound envelope are accurately encoded. However, the timing of AN action potentials will be triggered by (some subset of) the sharp flanks of the electric pulses. Fixed pulse rate CI sound encoding strategies therefore cannot accurately encode temporal features of incoming sounds in the temporal patterns of AN fiber discharges.

To be fair, when the CIS algorithm was invented, CIs were only ever fitted on one side, so supporting ITD discrimination was not on the designer's mind. Given that conveying signal envelopes for a handful of frequency bands can be sufficient to permit speech recognition [50, 51], the CIS strategy proved able to provide patients with the most important information, and it did so in a manner that was also a lot more energy efficient than prior analog coding approaches. Variants of CIS quickly became the dominant coding strategy, and they remain dominant today, over 30 years later. However, in recent years, bilateral implantation has become more common. In fact, in many countries it is routine at least for early deaf children. The demand for CIs is also growing rapidly in Asian markets like China, Thailand, or Vietnam, where tonal languages are spoken, which rely on voice pitch cues that are poorly encoded by CIS. These developments have made the question whether CIs should be redesigned to incorporate pulse timing (PT) codes for better binaural hearing and better pitch perception a hot, but also a controversial topic [24].

We should note that one CI device manufacturer, MED‐EL, has developed devices capable of offering “fine structure processing” strategies (FSP, FS4, and FS4‐p), which do encode the timing of temporal fine structure in the low frequency channels in the timing of electrical stimulus pulses. To achieve this, the biphasic stimulation pulses are temporally synchronized with the negative zero crossings of the wave form of the band‐passed acoustic signal [48]. However, this is done only for a small subset of up to four apical stimulation channels. This provision of fine structure information to at most four low‐frequency intracochlear channels contrasts with the AN responses in normal hearing, where, as we had seen in Figure 2, fine structure information is encoded in a highly coordinated and consistent manner across the entire frequency range. Furthermore, the timing of the zero‐crossing triggered pulses is tied to an internal clock which runs at relatively modest sampling rates of between 3 and 10 kHz. This limits the precision of the timing of these pulses to a range of ±333 to 100 µs, respectively [52]. In our view, more precise temporal coding across a wider range of frequency channels may be necessary to take full advantage of the auditory system's capabilities to process temporal codes.

## 5 Pitch Sensitivity in CI Patients

The auditory quality that makes some sounds sound higher or lower than others is called “pitch”. A simplistic introduction to the science of hearing may give a very misleading impression that pitch is a simple phenomenon. When sine waves increase in frequency, the place on the basilar membrane they maximally stimulate shifts from apical to increasingly basal positions, and the pitch they invoke increases at the same time. So, frequency maps onto a tonotopic place code, which in turn maps onto pitch, right? Well, we saw in Figure 2 that this view is hopelessly simplistic, not least because almost all sounds of interest are composed of multiple, often very many, frequency components, which stimulate the tonotopic place codes at many places at once. At the same time, the place code has, as we have seen above, limited frequency resolution, with maybe only as few as 40 fully independent frequency bands across the whole audible frequency range. Also, frequency resolution gets worse (cochlear filters get broader) with increasing frequency. The artificial vowel stimulus in Figure 2 had a fundamental (“repetition”) frequency of 80 Hz, meaning that it would be composed exclusively of integer multiples (“harmonics”) of the fundamental: 80, 160, 240, 320, 400, 480, … Hz. To determine that the pitch of this sound is 80 Hz from the place code, we would need to see peaks of high activity for fibers tuned to these harmonics, separated by troughs of noticeably lower activity for frequencies between the harmonics (120, 200, 280, 360, … Hz). Such peaks and troughs are not at all obvious along the characteristic frequency y‐axis of the AN fiber discharges shown in Figure 2. In the parlance of auditory physiology, the harmonics are not at all well “resolved” by the cochlear place code, in contrast to the temporal pattern code, which, as we have already mentioned earlier, encodes the 80 Hz pitch period beautifully in periodic bursts of action potentials.

So does this mean that pitch is all about temporal coding rather than place coding? Not so fast. Figure 2 shows responses of only a small subset of the AN fibers that the brain would get to see, and if we could observe many more fibers, we might be able to see that the low frequency, apical, end of the cochlea can indeed resolve some of the lowest harmonics through place coding. Based on a large body of psychoacoustic experiments summarized in clear and comprehensive reviews by Oxenham [15, 23], many researches studying pitch have come to believe that these “resolved harmonics” at the low frequency end of the cochlea do constitute an important pitch cue for many normally hearing listeners. These place cues are likely combined with temporal cues to create a final pitch percept. However, it remains unclear how and where this combination happens, how much it is shaped by experience, and whether all listeners end up with the same “recipe” for combining place and temporal cues to create their perception of pitch.

Where does all this leave CI listeners? In theory, CI electrodes ought to be able to access both place and timing codes for pitch, but at present they access neither of these well. It has been known for a long time that one can increase the perceived pitch of an electric pulse train delivered through a CI either by increasing the pulse rate or by delivering the pulse train to a more basal electrode position. However, an ingenious “multidimensional scaling” experiment on CI listeners has shown that pitch changes due to temporal cues are qualitatively different from pitch changes due to place cues [53]. CI users are often not particularly good at using either place or temporal cues, but they can improve with training [54, 55]. Interestingly, training CI users in the use of place cues does not improve their use of temporal cues, or vice versa [55]. In normal hearing, it is impossible to completely divorce place from temporal coding in the way it can be done in a CI, so we cannot experience what these “pitch qualities” sound like. However, given the aforementioned poor spatial resolution of CI place codes, delivering spatially resolved harmonics to their appropriate locations at the apical end of the cochlea is not a realistic goal [56]. We tend to imagine that stimulating different points along the cochlea with a CI may produce differences in “brightness”, similar to the way in which the ‘i’ in ‘sit’ sounds ‘higher’ than the ‘oo’ in ‘soot’.

CI users probably rely mostly on temporal cues to achieve pitch discrimination. Given that clinical devices use fixed pulse rates in most or all of their electrode channels, CI users will have to derive these temporal cues mostly from amplitude modulations of the pulse trains delivered through their CIs. As we have just seen in Figure 3, this constraint introduces inaccuracies in the temporal code present in the AN, but in addition it also limits the range of pitches that can be encoded through amplitude modulations. In signal engineering, the modulation rate must always be quite a lot lower than the rate of the carrier signal, because we need several cycles of the carrier to properly “fill out” the peaks in the modulation if these peaks are to be easily distinguished from the valleys. A CI processor running at, for instance, 1000 pps is therefore incapable of encoding a fundamental frequency higher than a few hundred Hz. Would CI processors that synchronize pulses to the fundamental frequency be able to encode fundamental frequencies in the kHz range? Sadly, they probably would not, because the maximum rate that is encodable with temporal coding through CIs is limited by another factor: “hyper‐synchronization”.

Hypersychronization describes at first glance the counterintuitive idea that CI stimulation may produce too much, rather than too little, synchronization between incoming stimuli and neural action potentials. In normal hearing, temporal features of incoming sounds are transduced by hair cells and transmitted to AN fibers via chemical synapses, which are somewhat imperfect transmission devices. Every inner hair cell connects to about a dozen AN fibers, and on every cycle of a periodic sound, only a subset of these nerve fibers will be activated. Furthermore, the random nature of the diffusion of neurotransmitters from hair cell to AN fiber adds some temporal jitter to the transmission of information. Each cycle of a periodic sound then triggers a temporally slightly diffuse volley of action potentials in a subset of the AN fibers. In comparison, electrical stimulation is a lot more precise and a lot more “all or nothing”. If an electrical stimulus pulse is large enough to trigger one AN fiber, then it is almost guaranteed to be large enough to also trigger the fiber right next to it, and it will do so at more or less exactly the same time. We therefore expect that electrical stimulation will lead to a temporally more precise and more synchronous activation of a larger proportion of AN fibers than comparable acoustic stimulation would, and recording experiments performed on cat AN confirm this [57].

To understand that this robust and precise synchronization can be “too much of a good thing”, we have to remember that, in normal hearing, the temporal code is carried by volleys of action potentials that are distributed across a variable subset of AN fibers on each cycle. This comparatively sloppy way of coding is advantageous because it makes it possible for nerve fibers to “sit out” a proportion of the stimulus cycles. This becomes a big advantage when stimulus cycles start to come in at a rate that is fast compared to the nerve fibers’ relative refractory period. It gives nerve fibers more opportunity to recover. Consequently, under acoustic stimulation, the AN appears to be able to phase lock at rates of a few kHz, even if the exact upper limit of phase locking in normal hearing is somewhat controversial [58]. In contrast, under electrical stimulation, the ability of AN fibers and brainstem neurons to keep up with stimulus pulses starts to decline dramatically when pulse rates exceed ∼ 300 to 500 Hz [57, 59]. This may be an important factor underlying the inability of CI users to process temporal pitch cues higher than a few hundred Hz, even if these are encoded through precise PT [60].

Given that presenting resolved harmonics through place coding in CIs appears unachievable, and with temporal coding limited to a few hundred Hz even under the best conditions due to hypersynchronization, some researches are rather pessimistic about the prospects of improving the pitch perception that may be attainable by CI uses. However, there are studies that give reason for optimism. First, work by Goldsworthy and Shannon [54] indicates that CI users who are provided with precise temporal pitch cues though experimental processors will learn to become progressively better at using them, which suggests that current standards in clinical processing strategies and rehabilitation practices does not give CI patients pitch discrimination abilities that are as good as they could be. Second, there are reports that the use of fine structure processing strategies with better temporal coding may help speakers of tonal languages like Chinese to perform better in the important pitch‐related task of lexical tone discrimination [61]. We therefore believe that research aiming to improve pitch discrimination through better CI coding strategies could well lead to significantly better hearing outcomes for many patients.

## 6 ITD Sensitivity in Bilateral CI Patients

Given the limitations in temporal coding in CIs that we discussed earlier, it will come as no surprise that ITD sensitivity in bilaterally implanted CI patients is generally substantially worse than in normally hearing controls. What may be more surprising is that the ITD sensitivity of CI patients remains poor even when tested with experimental processors that bypass CIS and deliver ITDs encoded in pulses timed with microsecond precision [41, 62, 63, 64]. Patients with early hearing loss show the poorest ITD sensitivity [65, 66, 67]. This is illustrated, for example, by Litovsky et al. [65], who reviewed data from a cohort of 34 bilateral CI (biCI) patients with different onset times of deafness. The poorer ITD sensitivity observed in prelingually deaf CI patients was later confirmed by the study by Ehlers et al. [66] testing 10 prelingually deaf children. We replotted their data here in Figure 4. All symbols in Figure 4 show behavioral ITD sensitivity thresholds measured with precisely timed electrical pulse train stimuli delivered to electrodes in the middle turn of the cochlea of each ear. The circles show the ITD thresholds of CI patients who lost their hearing either in adulthood (green circles), childhood (blue circles), or prelingually (red circles). For comparison, the dotted purple line shows the approximate ITD threshold one would expect to see in normally hearing humans [36, 37, 39, 68].

**FIGURE 4:**
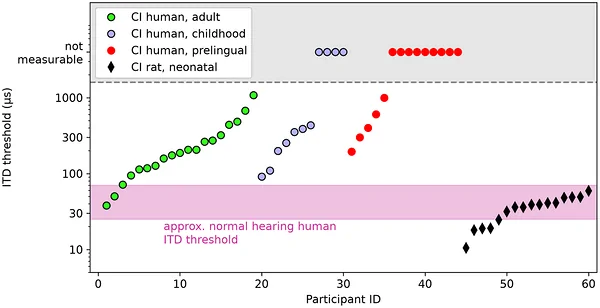
Interaural time difference (ITD) discrimination thresholds for 44 human CI patients (circles) with varying degrees of hearing experience, as well as for 16 neonatally deafened CI rats (diamonds). Participant IDs are sorted by ITD threshold within each cohort. The bilateral CI patients lost their hearing either in adulthood (green circles), in childhood (blue circles), or very early in life (prelingually, red circles). The testing was performed using research processors that deliver precise pulse timing ITDs. Unlike the human patients, the 16 neonatally deafened, biCI rats (black diamonds) had no prelonged exposure to CIS‐based stimulation with inaccurate pulse timing ITDs. The horizontal pink bar shows the approximate range of ITD thresholds reported for normally hearing humans. Human data from [65] and [66]. Rat data from [70].

Figure 4 shows that the large majority of biCI patients exhibit substantially elevated ITD sensitivity thresholds compared with the range typically observed in normally hearing people. The biCI patients deafened in adulthood shown in Figure 4 have a mean ITD threshold of approximately 270 µs. The median threshold across all biCI patients in Figure 4 was 258 µs. This means that the spatial resolution of this binaural cue is on average approximately between *four and ten times worse* than in normally hearing humans.

Figure 4 also shows that CI patients who became deaf early in life, either as children (blue circles) or as babies (red circles), frequently have particularly poor, or even absent, ITD sensitivity. In the study by Litovsky et al. [69], 4 out of 11 patients who became deaf in childhood, and 2 out of 4 patients who became deaf prelingually, had no detectable ITD sensitivity. Likewise, Ehlers et al. [66] reported that 7 out of 10 prelingually deaf patients had no measurable ITD thresholds within the tested range (≤ 1600 µs). In contrast, none of the 19 adult biCI patients of Litovsky et al. [65] (Figure 2, green circles) exhibited ITD thresholds > 1600 µs. This consistently impaired performance in the simple but important temporal processing task of ITD discrimination is not what one would expect if the temporal coding inaccuracies described in Figure 3 were the only factor at play, because all of the data shown in Figure 4 were collected with experimental stimulation protocols that delivered very precisely timed ITDs in the timing of the CI stimulus pulses. These observations therefore raise two important questions: What additional mechanisms contribute to the poor ITD sensitivity observed in the large majority of CI patients, and to what extent can novel PT strategies overcome these limitations if stimulation with precise PT alone does restore normal ITD sensitivity?

The observation that early deaf CI patients often show poorer ITD sensitivity had led to the plausible hypothesis that early deafness may result in a malfunction of the binaural timing circuitry of the auditory brainstem. According to the “critical period hypothesis”, a lack of auditory input during early development may prevent the normal development of these brainstem circuits [65, 66, 71, 72, 73]. However, while the human data reproduced in Figure 4 are indeed strongly suggestive of experience dependent plasticity playing a role, it clearly cannot be the only factor at play, given that adult deafened cohorts also exhibit greatly elevated ITD thresholds on average [65, 74, 75, 76, 77, 78].

Another factor that may limit ITD discrimination in biCI users is the hypersynchronization we had already described in the previous section. Hypersynchronization explains why ITD discrimination tends to become substantially harder at high pulse rates, but modeling work by Mueller and colleagues [59] further suggests that excessively precise synchronization of action potentials could hinder ITD processing even at lower pulse rates. Their modeling work suggests that ITD tuning curves of LSO neurons in the auditory brainstem should become excessively steep in this hypersynchronous regime, which presumably would make discriminating ITDs near zero (that is, very close to the mid‐sagittal plane of the listener), substantially easier, but making ITD discrimination away from the midline very much harder. To us it seems unlikely that hypersynchronization provides a comprehensive explanation of the poor ITD sensitivity seen in many biCI patients because this poor sensitivity can manifest also in simple left‐right ITD lateralization tasks performed at low pulse rates (e.g. [65, 66]), where the modeling by Mueller and colleagues [59] would not predict impaired performance. However, at the relatively high rates that contemporary clinical CI processors typically operate, hypersynchronization could certainly become an issue.

It is quite possible that multiple different factors conspire to impair ITD sensitivity in biCI patients, but unfortunately, our ability to investigate what is really going on here in human volunteers is very limited. For example, we hypothesized that the mechanism driving the poor ITD sensitivity seen in essentially all CI patients may have less to do with an absence of binaural input during early development, and more with the prolonged exposure to inappropriate PT information that bilaterally implanted CI patients experience every day as they listen to their environment with CIS‐style processors. As we have seen in Figure 3, the timing of CI pulses does not line up precisely with the temporal features of incoming sounds. So during normal everyday use, the PT ITDs experienced by these CI patients are not just uninformative, but also potentially highly misleading. The problem here is that our auditory system has evolved, not only to expect to receive useful ITD information, but also to then combine ITDs with other cues, such as interaural level differences (ILDs), to arrive at optimized overall estimates of the sound source direction. But if the quality of the ITD information received is consistently degraded, then incorporating these degraded ITDs could easily lead to worse, rather than better, final estimates of the direction that a sound came from. Consequently, the auditory pathway may react to a sustained “diet” of inappropriate PT ITDs by becoming desensitized to what would normally be a powerful binaural cue. Furthermore, this desensitization could be particularly pronounced in early deaf CI patients, who rarely or never experienced periods during which ITDs were consistently accurate and useful. Unfortunately, it is essentially impossible to test these ideas in human patient volunteers, given that they clearly need to continue using the currently approved processing strategies for many hours a day to benefit, for example, from the valuable speech recognition cues that their devices do provide.

To achieve good speech recognition and learn to navigate through the auditory environment, CI patients typically have to invest many months of practice, during which they learn to make the most of the specific, and quite unnatural, input provided by their particular devices. In doing so, learning to ignore and discard uninformative patterns is likely just as important as learning to recognize and interpret informative patterns. For the patients, this is an important journey that allows them to make the most of the currently available standard of care, but for scientists trying to develop novel CI coding strategies, this creates a massive confound that is extremely difficult to work around. If you ask a patient who has spent months learning to listen to the world through CIS‐based strategies to listen to sounds encoded with a novel, radically different research strategy, the most likely outcome will be that the patient's auditory pathway will find the strategy so unfamiliar that it won't know what to make of it. A fair comparison of novel, PT based strategies against CIS‐style strategies in current clinical use would require that patients be given the opportunity to use and to practice with the new strategy for long periods of time. The fact that performing studies like this would be technically very difficult, as well as slow and expensive, is at present probably the biggest obstacle to the development of novel processing strategies.

However, there are nevertheless some indications that routine exposure to fine structure information through CI processors may be very beneficial for ITD perception with bilateral CIs. These come from so far a limited number of quite small studies on CI patients, which use the above‐mentioned fine structure coding strategies from MED‐EL in their daily lives. Eklöf and Tideholm [79] studied a cohort of early deafened, CI‐supplied children, and found that 50% of the patients who used an FS strategy had ITD sensitivity with thresholds well below 1000 µs. In contrast, CI children who did not use a FS coding strategy showed no ITD sensitivity. Similarly, Fischer et al. [80] reported improved ITD thresholds when postlingually deafened CI patients were stimulated with the FS4 strategy, as opposed to when alternative strategies were used, which do not encode ITDs in PT. Nevertheless, the ITD or sound localization benefits that have been found in patients using FS strategies so far tend to be very modest. For example, Ausili et al. [81] reported that some patients with FS encoding were able to “exploit weakly informative temporal cues” to improve their sound localization, but the effect was small, and given the very small size of the control cohort without FS in that study (*N* = 5), as well as the large heterogeneity within and between groups, not large enough to reach statistical significance. Thus, the number of studies conducted to investigate potential benefits of MED‐EL's FS strategies remains quite small, recruiting large cohorts to achieve high statistical power remains difficult, and the benefits that have been described so far appear to be somewhat modest at this time, leading some to be skeptical whether temporal fine structure coding in CI can really be a game changer. However, as yet unpublished data collected at our Freiburg lab on CI patients show very robust binaural hearing benefits from FS coding in a task that isolates the low frequency channels very precisely.

Furthermore, as we had mentioned above, the current FS4 strategy differs from natural hearing in that PT information is provided with somewhat limited precision to a minority of frequency channels, and it is possible that making more precise PT information available to the full range of frequency channels would yield larger benefits. This idea is supported, for example, by data from Egger and colleagues [82], which show that delivering congruent ITD cues over two widely separated channels simultaneously can lead to better ITD sensitivity than delivering the cue to only one channel. Similarly, Kan and colleagues [83] report that delivering ITDs consistently along the whole electrode array produces better sensitivity to ITD than restricting stimulation to only the ends of the electrode array. In the next section, we will turn our attention to a wealth of compelling data from animal research that now supports the idea that very precise temporal coding can be highly beneficial for spatial hearing with biCIs.

## 7 Improving Spatial Hearing With biCIs: Latest Findings From Animal Research

To work around the many limitations inherent in clinical studies with human patients, we have developed a behavioral animal model, which allowed us to investigate PT ITD sensitivity during CI stimulation while fully controlling our animals’ acoustic or electric hearing experience. After demonstrating that normally hearing rats can learn easily and quickly to lateralize ITDs with respectably low thresholds of ∼50 µs [84], we raised neonatally deafened (ND) rats to young adulthood, fitted them with biCIs, and then trained and tested them on essentially identical ITD lateralization tasks [85, 86, 87, 88]. The results were remarkable. The black diamonds shown in Figure 4 demonstrate the behavioral ITD thresholds of 16 ND biCI rats tested with 300 pps pulse trains [70]. The mean ITD discrimination threshold for these animals is only 35 µs, comparable to the thresholds typically seen in normally hearing humans, and not significantly different from those of normally hearing rats (sign rank test compared to comparable data from [84], *p* = 0.16). The mean ITD threshold for these 16 ND biCI rats is thus almost *eight times* better than the mean threshold for the adult biCI patients shown in Figure 4. None of our biCI animals had an elevated ITD threshold, even though they received no auditory input throughout their development until sexual maturity.

Our animal data are both surprising and highly encouraging, as they show that essentially normal ITD discrimination is at least in principle achievable through biCIs, even after prolonged hearing impairment throughout early life. This may seem unexpected also in light of previous electrophysiological studies on congenitally deaf cats and neonatally deafened rabbits [89, 90, 91, 92, 93, 94, 95], which reported comparatively poor ITD tuning in auditory midbrain or cortex neurons of these early deaf animals. However, a careful look at these studies shows that the authors scanned neuronal ITD sensitivity in very large steps and tested very few ITD values small enough to fall within the physiological range of the animals. Most of the ITDs tested were too large to be of much use when one tries to predict an animal's likely ability to distinguish ITDs as small as a few tens microseconds. We therefore conducted new recording experiments, measuring ITD tuning curves of inferior colliculus (IC) neurons in biCI rats (Figure 5A) with and without early hearing experience, in which we focused on sampling the animals’ physiological ITD range in small, 20 µs steps [96]. These electrophysiological measurements were performed under anesthesia, within hours after implantation, so that there was essentially no opportunity for experience‐dependent plasticity to shape the observed neural tuning. The responses observed in the ND rats are therefore indicative of the “raw” state of an auditory pathway that has grown up without any experience of either acoustic or prosthetic auditory stimulation. Even in this “raw”, inexperienced and developmentally deprived state, 85% of the recorded multiunits (groups of neurons) showed sensitivity to ITD in the range of ±160 µs. This is very similar to the 83% of ITD sensitive multiunits detected in normally hearing experienced auditory midbrains. In both hearing deprived and hearing experienced rats, many multiunits significantly modulated their firing rates in response to ITD changes of only a few tens of microseconds, as shown in the examples in Figure 5B,C. Figure 5C shows an IC multiunit in an ND CI rat whose firing rate halves when the ITD changes from near zero, ±20 µs ITDs indicative of a source location near the midline, to a more lateral direction marked by an ITD as small as +60 or −60 µs. These data thus demonstrate that the auditory midbrain even of entirely naive ND animals is capable of encoding abundant amounts of information about PT ITDs with a resolution of tens of microseconds. Additionally, they explain why it is not difficult to train ND CI animals to lateralize even very small PT ITDs accurately, as demonstrated in [70, 85, 87, 97].

**FIGURE 5:**
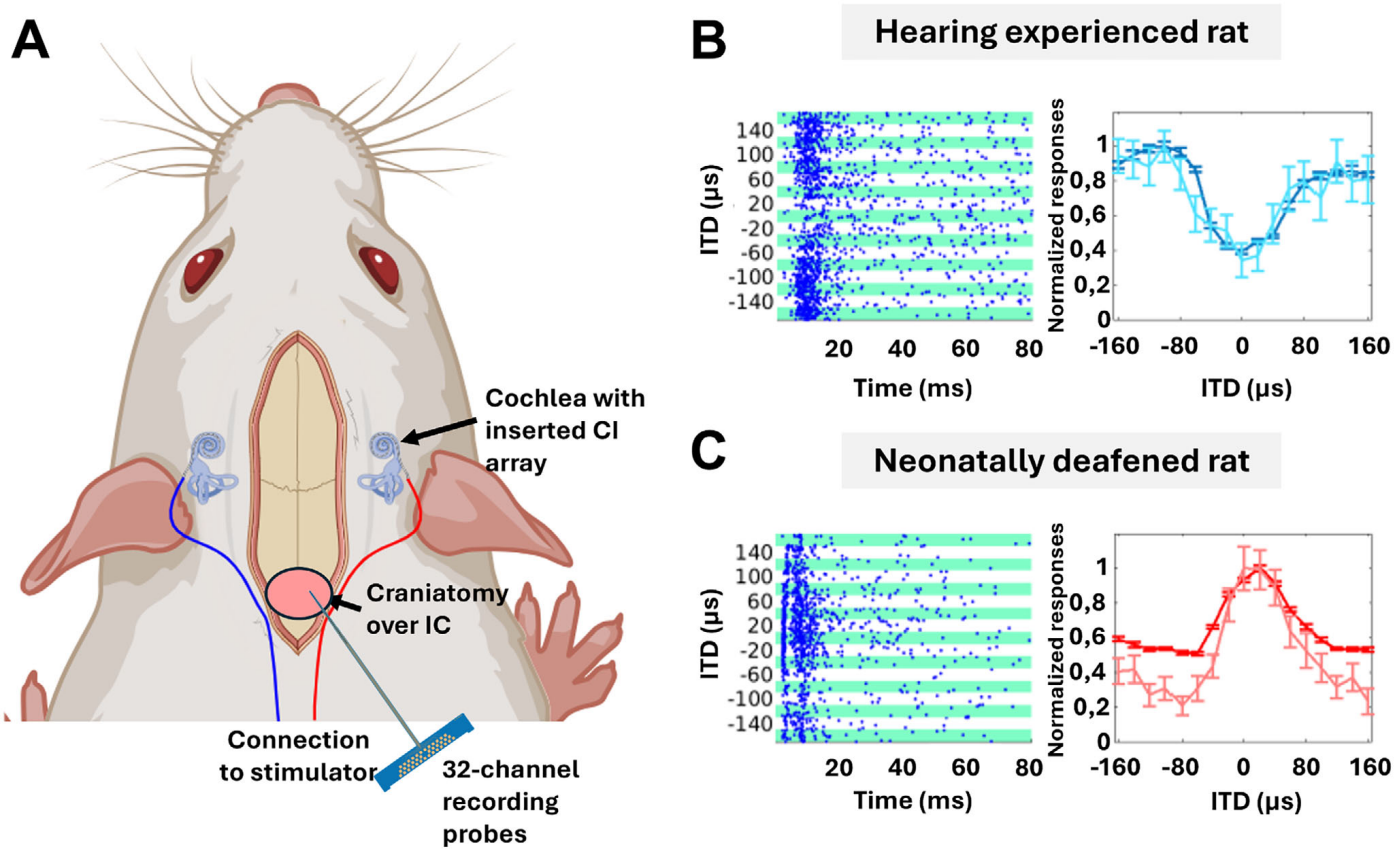
ITD sensitivity of neurons in the ICs of hearing experienced and neonatally deafened biCI rats. (A) Schematic representation of a CI rat during electrophysiological measurements. The craniotomy was performed above the ICs, and the extracellular responses of IC multiunits were recorded using a 32‐channel recording probe. (B+C) Left: Spike‐triggered activity diagrams and tuning curves for representative individual multiunits from a hearing‐experienced (B) and a neonatally deafened (C) CI rat. While both example multiunits exhibit clear ITD sensitivity, they differ in their preferred ITDs, illustrating the variability in ITD tuning observed across the two cohorts. Each blue dot represents a spike, with each stimulus repeated 30 times, thus, consecutive dots indicate a response to repeated stimulus presentation. Neural responses to different ITDs are shown alternately in green and white stripes. Right: Tuning curves for the corresponding multiunit. The error bars show the SEM value of the response amplitude at 30 presentations of each ITD stimulus. The dark blue and dark red tuning curves were determined from the amplitude of the analog multiunit activity responses, while the pink and light blue tuning curves were calculated from the simple threshold crossing of multiunit spikes. The tuning curves were baseline‐corrected and normalized relative to the maximum response. Negative ITD values indicate leading pulses contralateral to the recording site. The schematic illustration of the rat in (A) was generated using BioRender.com. (B, C) are adapted from [96] with permission.

Our observation that biCI rats are able to distinguish very small ITDs has opened up new experimental avenues for investigating factors involved in the development of ITD sensitivity. We have already mentioned the possible influence of a “critical period” in early development. This now appears to be less important, as the excellent ITD sensitivity shown in Figures 4 and 5 was observed in biCI rats without hearing experience during their early development. Of course, humans develop much more slowly than rats, and early deaf patients may undergo much longer periods of auditory deprivation in absolute terms than our ND rats did, so one may not wish to completely discount the potential involvement of critical period effects in human CI patients based on our rat data alone. Nevertheless, our data that biCI rats are as good as normally hearing ones in ITD discrimination, dramatically better than human biCI patients, irrespective of hearing experience, gives a tantalizing demonstration that much better outcomes in human patients might well be achievable if only we had a better understanding of the factors that currently limit the performance of human CI users in temporal processing tasks.

To elucidate other factors that might contribute to the reduced ITD sensitivity in CI patients, we have used our behavioral biCI rat model to answer three important questions: (1) Do ITDs have to be delivered through PT to be effective, or could pulse train envelopes be just as effective at delivering sub‐millisecond temporal cues? (2) How does ITD sensitivity interact with other spatial cues such as ILDs in the prosthetically stimulated auditory system, and how strong are ITDs relative to ILDs initially? (3) Provided that PT ITDs are indeed important, are the ITDs of all pulses in a train equally important? Or could it be that the CI stimulated auditory system, like the normally hearing one, places special emphasis on the first pulse in a brief pulse train or burst?

## 8 What is the Evidence That ITDs Should Really be Encoded in CI Pulse Timing?

Studies on CI patients investigating the relative effectiveness of PT ITD compared to envelope (ENV) ITD had already been conducted [74, 98, 99, 100]. However, given the limitations faced by human studies described above, these studies were necessarily restricted to biCI patients with measurable ITD sensitivity, whose auditory system had already adapted to prolonged clinical processor use. With our ND biCI rat model, however, we were able to investigate the relative effectiveness of PT and ENV ITDs in an auditory pathway that has not undergone prolonged exposure to clinical processor stimulation, using ITD values that are indicative of good binaural sensitivity (±80 µs), and at high pulse rates (900 and 4500 pps) [97]. A total of eight ND rats were fitted with biCI in young adulthood and, as before, trained to lateralize ITDs using a two‐alternative forced choice task (Figure 6A). During initial training, the animals experienced CI stimuli in which PT ITD and ENV ITD always agreed, so there was no ambiguity about which side would be a correct response deserving of a water reward. All electric stimuli were presented at pulse rates of either 900 or 4500 pps, using six different envelope types differing in envelope width (200, 50, 10 ms; Figure 6C). After the initial training, we introduced stimuli in which PT and ENV ITDs were allowed to vary independently, including “probe trials” (1 in 6 trials chosen at random), in which PT ITD and ENV ITD values pointed in opposite directions (Figure 6B). In such probe trials, the animals were rewarded irrespective of the spouts they chose. By analyzing the animals’ responses to a large number of stimuli with different PT and ENV ITD combinations, we were then able to quantify how strongly PT ITDs and ENV ITDs, respectively, can inform sound location judgments in the biCI stimulated auditory system.

**FIGURE 6:**
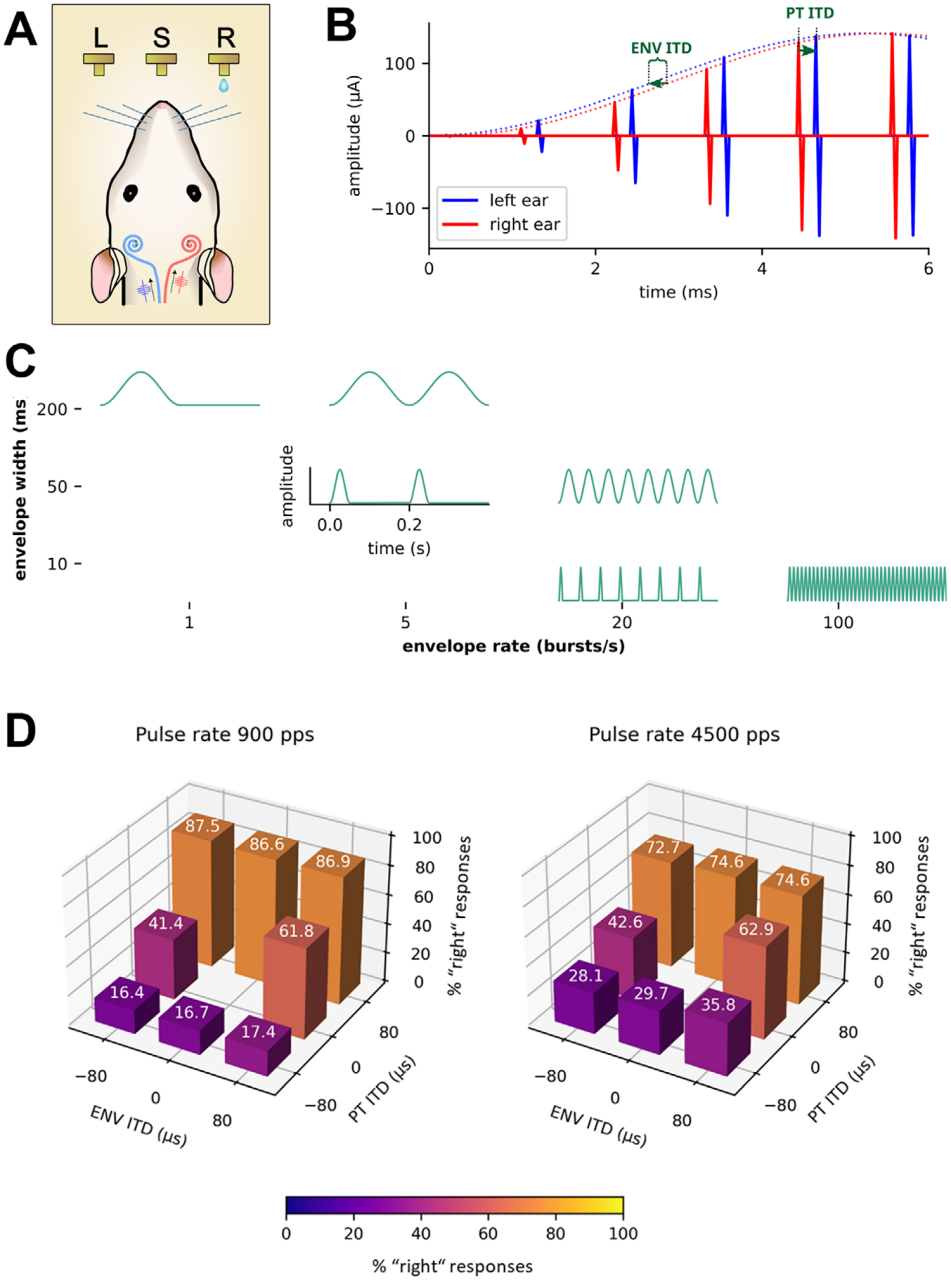
Pulse timing (PT) vs. envelope (ENV) ITD sensitivity in biCI rats. (A) Cartoon illustrating the behavioral paradigm. Rats were trained to initiate trials by licking a central start spout (“S”), triggering delivery of electric pulse trains with a small ITD to the left (blue) and right (red) ears. To obtain a water reward, animals had to lick the left (“L”) or right (“R”) response spout corresponding to the ear receiving the earlier stimulus. In the example shown, the right ear leads. (B) Schematic of the binaural pulse trains used. Biphasic pulse trains were amplitude‐modulated with Hanning window envelopes. ENV ITD and PT ITD were varied independently. In the example, the left ear envelope rises earlier than the right ear envelope, whereas left ear pulses occur later than right ear pulses. (C) To test whether ENV ITD sensitivity depends on envelope shape, six envelope types were used, varying in width (200, 50, or 10 ms) and the presence or absence of silent gaps (envelope rate equal to or greater than 1/envelope width). (D) 3D bar plots summarizing the proportion of “right” responses for two pulse rates (left: 900 pps; right: 4500 pps). White numbers above bars indicate the percentage of “right” responses for each combination of PT ITD (*x*‐axis) and ENV ITD (y‐axis). Figure adapted from [97].

The behavior data showed that all ND biCI rats were many times more sensitive to PT ITDs than to ENV ITDs, irrespective of pulse rate (Figure 6D) or envelope shape [97]. This stronger weighting of temporal fine structure information is also observed in normally hearing listeners: ITD thresholds for fine structure are substantially lower than those for ENV ITDs, while the combination of both cues generally produces sensitivity comparable to that obtained from fine structure ITDs alone [101]. Thus, the predominance of PT ITDs observed in our rats is consistent with the relative weighting of fine structure and ENV ITDs in acoustic hearing, although both cues can contribute to binaural lateralization when available [101, 102].

Focusing on the behavioral data at a clinical stimulation rate of 900 pps (Figure 6D, left), it can be seen that a change in PT ITD strongly influenced the lateralization decision of our biCI rats. When the PT ITD was +80 µs (right ear leading), the animals made on average ∼ 87% responses to the right, but when the PT ITD was −80 µs (left ear leading), the mean proportion of responses to the right fell to only ∼ 16.8%. In comparison, changing the ENV ITD had a much smaller effect on the rats’ decisions. Particularly for nonzero PT ITDs, %right responses barely change at all with changing ENV ITD.

An obvious question is how PT can favor ITD discrimination even at such high pulse rates, especially since one would expect that individual pulses cannot be resolved at rates above 4000 pps. One possible answer to this question is that at high pulse rates, the auditory brainstem generates an onset response when the first pulse in the stimulus sequence is large enough to elicit a neural response in the AN. The results of animal experiments published by Hancock et al. [103] support this hypothesis. In that case, the onset responses that the brainstem uses to time envelope features are still “locked to the temporal grids” set up by the PT generators in each CI processor. PT exerts a significant influence on temporal processing, even at pulse rates so high that the auditory brainstem is no longer able to represent each pulse in a train individually. In any event, these data show that the timing of individual pulses matters, and that it cannot be substituted with precise envelope timing. To further support this hypothesis, the next critical step will be to conduct chronic experimental studies that directly compare biCI animals exposed to CIS‐like ENV ITD only stimulation with biCI animals that receive informative PT ITD cues.

The decision by CI companies to use encoding strategies in which the PT is largely or completely independent of the temporal characteristics of the incoming sound therefore very likely leads to significant limitations in the audio quality that these prosthetic devices can deliver. Recent modeling work by McDermott's group further supports this idea. By simulating possible causes for the poorer hearing performance of CI patients in neural network models of CI‐mediated hearing, they provide further evidence that the usual clinical provision of temporal information exclusively on the envelope of the electrical stimulation is bound to be insufficient [104].

## 9 How Does the CI Stimulated Auditory Pathway Combine ITD and ILD Information, and How Strong are ITD Cues Initially?

A biCI patient with clinical processors currently receives essentially random pulse ITDs generated by the relative phase delay between the two pulse generators, which stimulate the left and right ears independently of each other [105]. For illustration, let us assume a pulse rate of 1000 pps per electrode. At this stimulation rate, the respective channels in each ear receive approximately one pulse every millisecond independently of each other, and the pulse ITDs are therefore random numbers between zero (pulses happen to be synchronized) and 500 µs (pulse in one ear happens to fall exactly in the middle between two pulses in the other ear). The phase relationship between the two processors is also not fixed, but drifts slowly and continuously in a manner that keeps PT ITDs unpredictable as well as uninformative [105].

Skeptical readers may object that our assumption here is unrealistic because clinical CI processors can be set to operate at pulse rates that are faster than 1000 pps, and that any one AN fiber may receive stimulation from more than one adjacent stimulating electrode. This would result in smaller inter‐pulse intervals in each ear, which in turn would reduce the range of random PT ITDs that can occur. With higher and higher pulse rates, the range of possible random PT ITDs would shrink, perhaps to the point that they can no longer inject troubling amounts of noise into the binaural hearing circuits of the auditory pathway. However, given the issues related to hypersynchronization previously described, we have good reason to believe that pulse rates of 1000 pps are already very fast relative to the limited ability of AN fibers to encode short intervals between electrical stimulus pulses. Recordings from bushy cells in the cochlear nucleus, which receive direct input from AN fibers, clearly document rapid declines in firing probability when electrical stimulation pulse rates increase above a few hundred pps ​ [59]. Therefore, one cannot simply shrink the range of possibly misleading PT ITDs by increasing pulse rates because high pulse rates will introduce more firing failures and thereby degrade the neural timing code in each AN.

Based on what we discussed in the previous section, we would expect that a newly implanted, still inexperienced auditory system will be highly sensitive to the random PT ITDs that CIS‐style processors will throw at it, but what would it do with them? In normal spatial hearing, ITD cues are unconsciously combined with ILDs and other cues to form an integrated perception of the source location. When ITD and ILD cues disagree, the brain normally tries to settle on a compromise localization estimate. For example, an auditory stimulus that is slightly louder in the left ear but slightly earlier in the right ear may be perceived as coming from the midline. This is a phenomenon known as “time‐intensity trading”, and the relative strength of ITD and ILD cues in shaping overall spatial perception is called the time‐intensity trading ratio (TITR), which is measured in µs/dB [106, 107, 108, 109]. To understand the likely impact of randomized PT ITDs delivered by clinical devices, it would be important to know TITRs for the CI‐stimulated auditory system in its native state. The more strongly TITRs are weighted in favor of ITDs (i.e., the smaller the TITRs), the greater the potential for random PT ITDs to disrupt spatial hearing in CI patients by overriding any useful ILD information the patients do receive from their bilateral implants.

We recently published a behavioral study in which we investigated the interactions between ITD and ILD cues in nine ND biCI rats [87]. Figure 7A,B illustrates the types of stimuli used in these experiments. Figure 7A shows examples of two “honesty trial” stimuli (∼ 80% of the cases) in which ITD and ILD do not point toward opposite sides, and Figure 7B visualizes one example of a “probe trial” stimulus (∼ 20% of the cases) in which ITDs and ILDs point in opposite directions. While the animals had to respond correctly to receive a water reward in an “honesty trial”, the animals were always rewarded for “probe trials”. In the experiment we tested a large set of ITD and ILD combinations, but to provide an easy to follow example of how even small changes of ITD cues can affect the overall resulting lateralization judgments, we show behavioral responses to just a subset of these stimulus combinations in Figure 7C. The figure shows the percentages of trials on which each animal indicated that it heard the stimulus on the right hand side. When a combination of ILD = −4 dB (left leading) and ITD = 0 µs (straight ahead) was presented, the animals responded on the right side on average 34% of the time. In other words, on two‐thirds of these trials they heard the sound on the left, and this is as expected given that the 4 dB left ear leading ILD should provide a noticeable cue pointing to the left‐hand side. However, when we kept the ILD at −4 dB but changed the ITD cue from 0 to +80 µs (right leading), this strongly affected the lateralization decisions of the biCI rats, who now responded on the right hand side in the majority (59%) of the trials. Remember from our earlier discussion that more than half of human biCI users shown in Figure 4 had thresholds above 250 µs, and only three of the 34 patients in Figure 4 had ITD thresholds below 80 µs. Nevertheless, here we see that an ITD as small as 80 µs can make biCI animals change their minds about where they heard a stimulus they had lateralized on the basis of ILDs.

**FIGURE 7:**
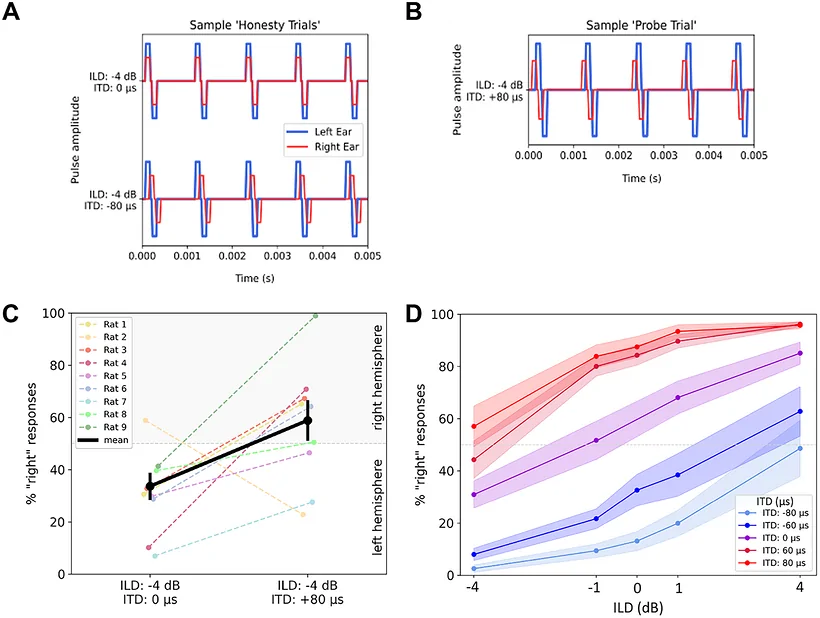
Time‐intensity trading ratio experiments in neonatally deafened biCI rats. (A) Two example “honesty trials”. The upper panel shows a binaural electric pulse train with an ILD of −4 dB and an ITD of 0 µs; the lower panel shows a stimulus with an ILD of −4 dB and an ITD of −80 µs. In both cases, rats had to respond at the left spout to receive a water reward, whereas incorrect responses resulted in a timeout. (B) Example “probe trial” with an ILD of −4 dB (favoring the left side) and an ITD of +80 µs (favoring the right side). During probe trials, rats were free to choose either response spout and were rewarded regardless of choice. (C, D) Percentage of rightward responses in nine CI rats across different binaural cue combinations. The *y*‐axis indicates the proportion of rightward responses; because only left or right choices were possible, values below 50% indicate a bias toward leftward responses. (C) Individual data from the nine rats for two cue combinations: ILD −4 dB/ITD 0 µs and ILD −4 dB/ITD +80 µs. Colors denote individual animals; the group mean is shown in black, with error bars representing the standard error of the mean (SEM). (D) Effect of ILD on lateralization judgments averaged across all rats for different ITDs (colored curves), with ILD shown on the x‐axis. Figure adapted from [87].

Figure 7D provides a more complete picture of the data collected in this study, and of how strongly and systematically ILD psychometric functions can be shifted by even quite modest changes in ITD. It shows the mean percentage of right responses across all biCI rats (± SEM as shaded areas) for all the different cue combinations tested. The x‐axis represents stimulus ILD, the *y*‐axis the observed percentage of “right” lateralization decisions. The purple curve represents the ILD psychometric function at ITD = 0 µs. The two red curves correspond to right‐leading ITDs and are shifted upward, reflecting an increased proportion of rightward responses. The blue curves correspond to left‐leading ITDs and are shifted downward. Overall, the analysis of our behavioral data demonstrates that ITDs are weighted heavily in our biCI rats’ localization judgments, which is reflected in TITR values that were typically in the range of 20 µs/dB or smaller.

Assuming that the mechanisms for extracting and combining binaural cues are essentially similar across mammalian species, what would a TITR of around 20 µs/dB imply for a prelingually deaf patient who has just been implanted with a pair of clinical processors? Given the observations described above, we would not only expect this patient's auditory system to be very sensitive to small PT ITDs initially, but we would also expect this ITD sensitivity to exert a strong influence when ITD and ILD cues are combined. By simply extrapolating a TITR of ∼ 20 µs/dB, one might predict that random pulse ITDs of, for example, 250 µs could interfere with informative ILDs of up to 12.5 dB. If we now also remember that the dynamic range of stimulus intensities that can be encoded in CI electric hearing is quite modest, perhaps no larger than 10 to 20 dB [9]. It therefore seems possible that the random PT ITDs that bilateral CIS‐style processors produce would confound even the largest ILDs that these devices can possibly deliver, which would prevent any form of effective binaural hearing unless or until the auditory pathway loses its initially highly pronounced sensitivity to PT cues. Admittedly, arguments based on linear extrapolation well beyond the range of available observations must always be treated with caution, but the data in Figure 7C,D nevertheless clearly demonstrate that even small “wrong” PT ITDs have the potential to mask quite clear and strong “correct” ILD cues. At present, it seems reasonable to assume that this situation also applies to human biCI patients whose auditory system is constantly bombarded with uninformative PT ITDs from their clinical processors. We therefore hypothesize that these patients must somehow “learn” to become insensitive to misleading PT ITD if they are to be able to derive any benefits from the usually informative ILDs delivered by their devices. The poorer ITD sensitivity typical of biCI patients may therefore be due to an adaptation process that involves the suppression of the binaural system's innate sensitivity to timing cues, because the information it collects is only disruptive under clinical stimulation. The poorer ITD sensitivity of early deaf patients, as described in Figure 4, may be due to the fact that these patients had particularly little experience of useful ITD input and were fitted with implants at a relatively early age, when the auditory pathway may still be more plastic, and learned to suppress the unhelpful ITD input. This process appears maladaptive in that it reduces the ITD sensitivity of the auditory system, which should actually be well developed but in this context only confuses the system. The hypothesis that prolonged exposure to uninformative PT ITDs may lead to a decline in ITD sensitivity is in principle testable experimentally in our behavioral rat model, and experiments aiming to do that are currently underway in our laboratories. Similarly, this hypothesis would lead to the prediction that CI patients should be more sensitive to PT cues for ITDs or pitch immediately after implantation than after prolonged exposure to their clinical processors. This prediction should be testable in principle, but this would not be an easy experiment in practice.

## 10 Pulse Timing Matters, but Do All Pulses Matter Equally?

As we have seen, the timing of stimulus pulses in CI stimulation can matter a great deal, and the perhaps somewhat cavalier approach with which CIS‐style strategies disregard PT appears overdue for a re‐evaluation. However, there remains a gulf between deciding that one probably should develop CI coding algorithms that exploit the power of PT codes in the auditory system, and know how exactly these algorithms should achieve this. CIs should, in our view, use PT to precisely encode more of the temporal features of incoming sounds, but they must do so in a manner that does not compromise place coding or sound level coding. Remember that CIS opted for fixed rate, stimulus independent pulse trains because this provided an easy and effective way to prevent any potential problems from channel interactions, which might occur when pulses sent at neighboring channels overlap in time. However, it is not very common that temporal features of natural sounds are “broad band”, and therefore occur more or less simultaneously in multiple frequency bands at once. A veridical representation of temporal features across the whole tonotopic array can therefore be difficult to reconcile with a prohibition of temporally overlapping pulses in different frequency channels. Furthermore, if the timing of stimulus pulses is determined by temporal features of the incoming sounds, then the density of those features, i.e., the number of features per stimulus time, directly influences the number of pulses that are delivered per unit time. However, if the number of pulses per second increases, then the stimulus will trigger higher firing rates in the AN and also be perceived as louder. We have a long way to go before we know how to make a perfect CI coding strategy that can deliver abundant temporal pattern information to a patient's AN and do so independently of loudness coding and also maintain good channel separation in place coding. As we try to progress toward that goal, one additional factor worth keeping in mind is that, in a train of stimulus pulses, not all pulses will be equally important or effective at driving neural responses or shaping auditory perception. An obvious example is that, when pulse intervals become short relative to neural refractory periods, the first pulse in a burst of pulses will be much more effective than subsequent pulses. Refractory periods of neural axons tend to last for no longer a few milliseconds, but the auditory system is also subject to “forward masking”, where relatively weak stimuli become inaudible if they occur within a period up to a few tens of ms after an intense stimulus. MP3 sound compression relies on spectral masking to make sound files smaller by removing the parts that are inaudible anyway because of masking. Perhaps somewhat analogously, incorporating an understanding of temporal masking into CI coding strategies may be helpful [110]. Binaural hearing too has effects where the properties of early pulses may, in a sense, mask the properties of later pulses. A specific example which we were able to examine with our biCI model is the so‐called “precedence effect” (PE). This effect stipulates that, when we try to localize a prolonged binaural stimulus, such as a rapid pulse train, our auditory system will base its localization judgment almost entirely on the initial pulse, the “first wavefront” of the sound, and largely ignore the binaural cue values of the later part of the signal. This is thought to be an adaptation for hearing in echoic environments, where the binaural cue values of later parts of a signal are bound to be distorted by echoes reverberating from all sorts of directions, and only the initial sound, which has reached our ear along the shortest path without bouncing off some echoic surface first, can be relied upon to contain binaural cue values indicative of the true sound source direction. Understanding how refractoriness, masking, the PE, and other phenomena influence the role and relative importance of each pulse in a neuroprosthetic pulse train stimulation paradigm may give useful clues to how to design better coding strategies in the future. We therefore set out to investigate whether the PE for ITDs in our biCI rats behaves in a manner that is entirely analogous to what is known from studies on normally hearing humans and animals.

To this end, we performed behavioral experiments to measure “temporal weighting functions” (TWFs), which quantify the PE by asking participants to localize brief bursts of binaural pulses, in which the binaural cue for each individual pulse in the sequence varies randomly and independently. Subsequently, a post‐hoc statistical analysis is performed to calculate how strongly the cue value of the first, second, … Nth pulse, respectively, influenced the participant's overall localization judgment. Brown and Stecker [111] used this approach to show that, in normal hearing humans, the binaural cues carried in the first pulse dominate, in that they have a much stronger influence on the perceived location of the entire burst of pulses than later pulses in the burst. This “onset dominance” becomes increasingly pronounced at higher click rates. We adapted Stecker and Brown's behavioral paradigm for the use in animals to test three different cohorts of rats: normally hearing (NH) acoustically stimulated animals (*n* = 4; [112]), neonatally deafened (ND) CI‐stimulated animals (*n* = 8), and adult deafened (AD) CI‐stimulated animals (*n* = 4)) [88]. For the acoustically stimulated animals we used trains of eight binaural, broad‐band (2–22 kHz) pulses delivered over tube phones. The ITDs for each pulse in the train were chosen uniformly and independently from the set {−120, −80, −40, 0, +40, +80, +120} µs [84, 112]. For the CI animals, the acoustic pulses were simply replaced by eight biphasic electric current pulses (duty cycle: 40.96 µs positive, 40.96 µs at zero, and 40.96 µs negative). All pulses had the same amplitudes. For NH rats, click trains were presented at an average binaural acoustic level of ∼ 80 dB SPL, while for CI rats, a stimulus intensity of 2–7.5 dB above each animal's eABR threshold was chosen. In order to monitor and control the animals’ behavior, the testing sessions randomly interleaved “honesty” and “probe” trials. Examples of the two trial types are shown in Figure 8A,B. In honesty trials, the ITD choice of the eight pulses in the stimulus was constrained such that they all had the same sign, that is, all ITDs pointed to the same side but still varied in magnitude. Each honesty trial, therefore, had only one objectively correct response and was rewarded only if animals responded accordingly. In probe trials, however, the ITDs for each click or pulse varied without constraint, and probe trials thus routinely contained pulses with both left‐ear leading and right‐ear leading ITDs. In order not to bias these choices during probe trials, probe trial responses were rewarded irrespective of which side was chosen. Using a Probit analysis to model the probability that the rat would respond on the right, we then computed the perceptual weight of each pulse. All three cohorts were tested at a stimulation rate of 300 pps (some cohorts also at other pulse rates). Consistent with previous studies in normal hearing humans, all NH rats exhibited a pronounced “onset dominance,” i.e., the weighting of the first pulse was much greater than that of the subsequent pulses (Figure 8C, blue). In contrast, the TWFs for the ND‐CI (Figure 8C, red) and AD‐CI animals (Figure 8C, yellow) were significantly flatter, with a reduction in initial dominance of approximately 75%, particularly at higher pulse frequencies [88].

**FIGURE 8:**
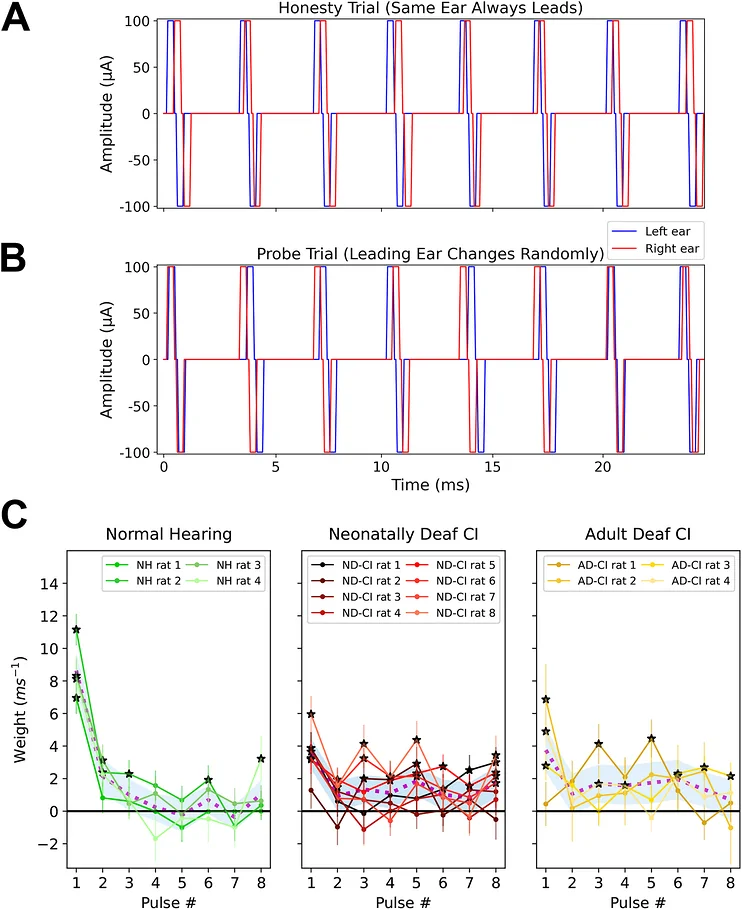
Precedence effect in acoustic and electric hearing rats. (A) Example “honesty trial” with only left leading ITDs and random jitter per pulse. Responses to honesty trials were only rewarded if the side of the leading pulse burst was chosen. (B) Example “probe trial” with ITDs jumping randomly between left and right ear leading. Responses to probe trials were always rewarded, independently of the animal's choice. (C) Temporal Weighting Functions (TWFs) for the normal hearing acoustic (green), neonatally deafened CI (red), and adult deafened CI (yellow) cohorts at 300 pps stimulation rate. Each dot shows the temporal weighting (probit regression coefficient in 1/ms) for each of the eight pulses within one burst. Asterisks show coefficients that are significantly different from zero (*p* < 0.05), circles show non‐significant coefficients. Error bars indicate SEM of individual coefficients. The magenta dotted lines show the mean across animals in each condition, and the blue shading shows the SEM across animals. Data from each animal are plotted in a different hue. Normal hearing acoustic data originally from [112]. Data in C previously published in [88].

Hearing in reverberant environments is a major challenge for CI patients, and our results strongly suggest that the development of a pronounced PE when listening with CIs may depend on highly specific experiences within this particular hearing modality. Prior exposure to echoic listening conditions during acoustic hearing, as in our AD‐CI cohort, does not appear to favor the development of pronounced initial dominance. We therefore hypothesize that listening experiences with CIs under echoic conditions are necessary for the auditory system of CI patients to exhibit strong PE, regardless of any normal binaural listening experiences prior to CI implantation.

The electrically stimulated auditory system appears to adapt in a very specific way to the reliability of binaural cue information in order to process and utilize incoming signals at different times. It remains to be seen whether future CI technology could be optimized to provide patients with sensory cues that enable them to better adapt to the unique challenges of hearing in reverberant environments using their auditory prostheses.

## 11 Conclusion

The preceding sections have painted a picture of CIs as both a remarkable success story and a work in progress. On one hand, these devices have restored meaningful speech perception to over a million patients worldwide—a feat that would have seemed like science fiction just a few decades ago. On the other hand, the quality of hearing that CIs provide remains stubbornly inferior to that of a healthy cochlea, particularly when it comes to music appreciation, tonal language comprehension, and spatial hearing in noisy or reverberant environments.

Where should researchers and device manufacturers direct their efforts to close this gap? Our analysis suggests that the most promising path forward lies in rethinking how CIs encode temporal information. For over thirty years, clinical devices have relied on CIS‐style strategies that deliberately decouple the timing of electrical pulses from the temporal fine structure of incoming sounds. This design choice was a reasonable engineering compromise at the time, as it minimized disruptive channel interactions and prioritized the faithful encoding of slow amplitude envelopes—the cues most critical for basic speech recognition. However, as we have seen, the AN in its native state is exquisitely sensitive to precisely timed patterns of action potentials, and the auditory brainstem uses these temporal codes for tasks ranging from pitch perception to sound source localization. By discarding precise PT, contemporary CIs may be leaving a wealth of useful information on the table.

Our animal model experiments offer three particularly compelling reasons to revisit this design choice. First, neonatally deafened rats fitted with bilateral CIs demonstrate ITD discrimination thresholds as low as 35 µs—indistinguishable from normally hearing animals and nearly an order of magnitude better than the average human CI patient. Second, these animals weight PT cues much more heavily than envelope timing cues when making lateralization judgments, with time‐intensity trading ratios around 20 µs/dB. Third, the auditory midbrain of even completely inexperienced, neonatally deafened animals contains abundant neurons capable of discriminating ITD changes as small as tens of microseconds. Taken together, these findings indicate that the poor ITD sensitivity observed in human CI patients is not an inevitable consequence of early deafness or of limitations in brainstem development. There could, of course, be a variety of different factors contributing to the generally poor ITD sensitivity seen in human CI patients, and more research is clearly needed, but our interpretation of the current data is that a learned, maladaptive response to prolonged exposure to random or misleading PT information delivered by clinical processors is one important factor at play.

If this interpretation is correct, then the path forward is both clear and challenging. It is clear in that it suggests that developing CI coding strategies that encode temporal features of sounds faithfully in the precise timing of stimulus pulses—rather than treating PT as an irrelevant nuisance parameter—could substantially improve outcomes for pitch perception and binaural hearing. It is challenging because such strategies must solve a difficult engineering problem: how to encode rich temporal patterns without exacerbating channel interactions or conflating timing cues with loudness. The clearest evidence of benefits from precise temporal coding comes from studies that used single‐electrode stimulation in each ear. Although existing human studies demonstrate that temporal cues can be preserved under multi‐channel stimulation, how best to integrate precise temporal coding with the spatial and spectral information provided by multiple electrodes remains an important challenge for next‐generation CI strategies. Moreover, introducing radically new coding strategies will require careful, long‐term studies to allow patients to learn to interpret unfamiliar temporal patterns, and regulatory pathways for such novel devices are neither fast nor inexpensive.

Nevertheless, the potential payoff is too large to ignore. The data from our animal model demonstrate that, in principle, nearly normal temporal processing is achievable through bilateral CIs, even after a lifetime of deafness. Realizing this potential in human patients will require a concerted effort from auditory physiologists, biomedical engineers, and clinical audiologists. It will also require a willingness to question long‐held assumptions about the relative importance of place vs. temporal codes in electrically stimulated hearing. If the field can rise to this challenge, the next generation of CIs may offer users something that remains elusive today: a truly rich auditory experience that captures not just the words people say, but also the music, the melody, and the sense of space that make hearing such a rich part of human experience.

## Author Contributions

**Nicole Rosskothen‐kuhl**: conceptualization, investigation, funding acquisition, writing – original draft, writing – review and editing, visualization. **Jan W. H. Schnupp**: conceptualization, investigation, funding acquisition, writing – original draft, visualization, writing – review and editing.

## Conflicts of Interest

The authors declare no conflicts of interest.

## Data Availability

Data sharing not applicable to this article as no datasets were generated or analysed during the current study.
